# Supplementary material for: Two-dimensional MXene membranes with biomimetic sub-nanochannels for enhanced cation sieving
Source: Nat Commun. 2023 Aug 15;14:4907. doi: 10.1038/s41467-023-40742-8 (PMC10427654; doi:10.1038/s41467-023-40742-8)
Supplement: Supplementary file 1 — Supplementary Information [file 41467_2023_40742_MOESM1_ESM.pdf]

Two-dimensional MXene Membranes with Biomimetic Sub-nanochannels for Enhanced Cation Sieving

Rongming Xu[1,2], Yuan Kang[3], Weiming Zhang[1,2]*, Bingcai Pan[1,2]*, Xiwang Zhang[4]*

[1] State Key Laboratory of Pollution Control and Resource Reuse, School of the Environment, Nanjing University, Nanjing 210023, China

[2] Research Center for Environmental Nanotechnology (ReCENT), Nanjing University, Nanjing 210023, China

[3] Department of Chemical and Biological Engineering, Monash University, Clayton, VIC 3800, Australia

[4] UQ Dow Centre for Sustainable Engineering Innovation, School of Chemical Engineering, The University of Queensland, St Lucia QLD 4072, Australia

* Corresponding authors:

wmzhang@nju.edu.cn; bcpan@nju.edu.cn; xiwang.zhang@uq.edu.au

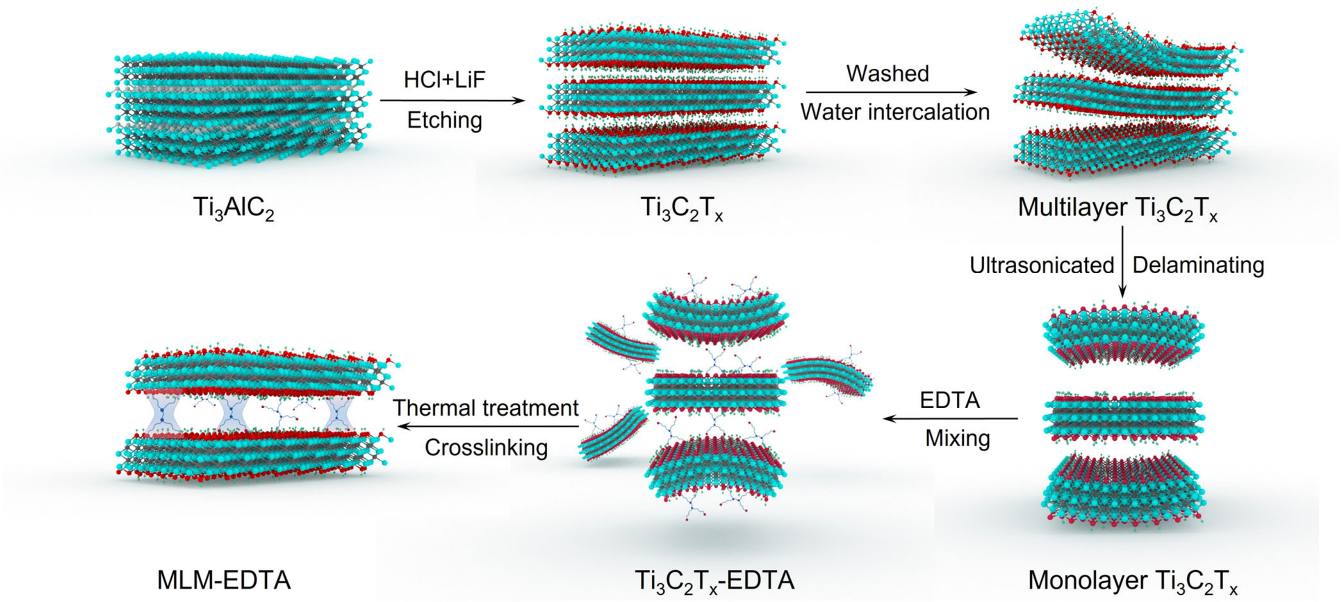

Supplementary Fig. 1 Schematic showing the preparation of MLM-EDTA.

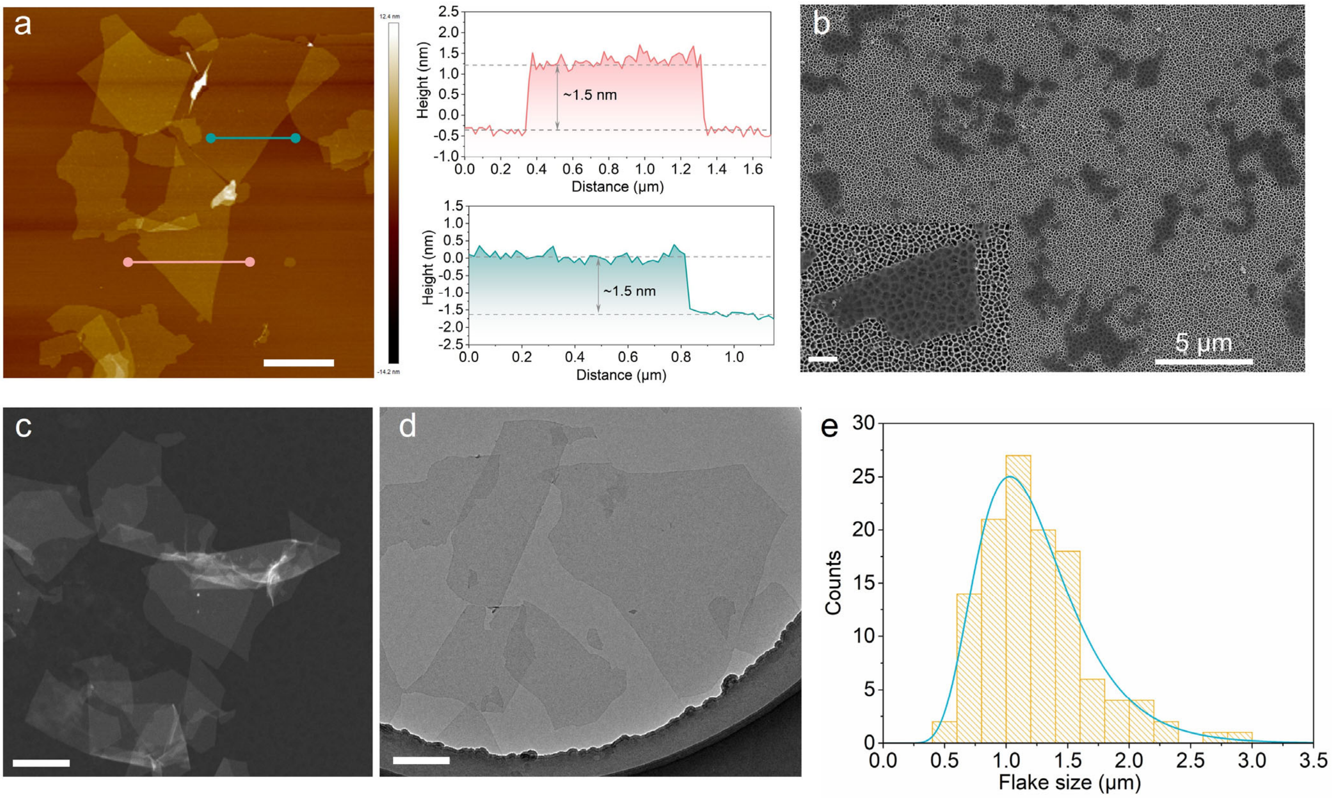

Supplementary Fig. 2 Characterization of $Ti_3C_2T_x$ nanosheets. (a) AFM image and corresponding height profiles of the $Ti_3C_2T_x$ nanosheet on mica disc. Scale bar, 1.0 μm. (b) SEM image of $Ti_3C_2T_x$ nanosheets deposited on AAO substrate. Scale bar, 5 μm. Inset: high magnification SEM image of a $Ti_3C_2T_x$ nanosheet. Scale bar, 500 nm. (c) High-angle annular dark-field (HAADF) transmission electron microscopy (TEM) image of $Ti_3C_2T_x$ nanosheets. Scale bar, 500 nm. (d) TEM image of $Ti_3C_2T_x$ nanosheets. Scale bar, 500 nm. (e) Flake size distribution of $Ti_3C_2T_x$ nanosheets.

TEM and SEM images show that as-synthesized $Ti_3C_2T_x$ nanosheets feature flat and wrinkle-free surface, with an average lateral size of around 1.0 μm. The as-synthesized $Ti_3C_2T_x$ nanosheets also have a thickness of about 1.5 nm, which is slightly larger than theoretically predicted value of 1.0 nm for monolayered $Ti_3C_2T_x$ nanosheet. This can be attributed to impurities adsorbed on the surface, such as water molecules.

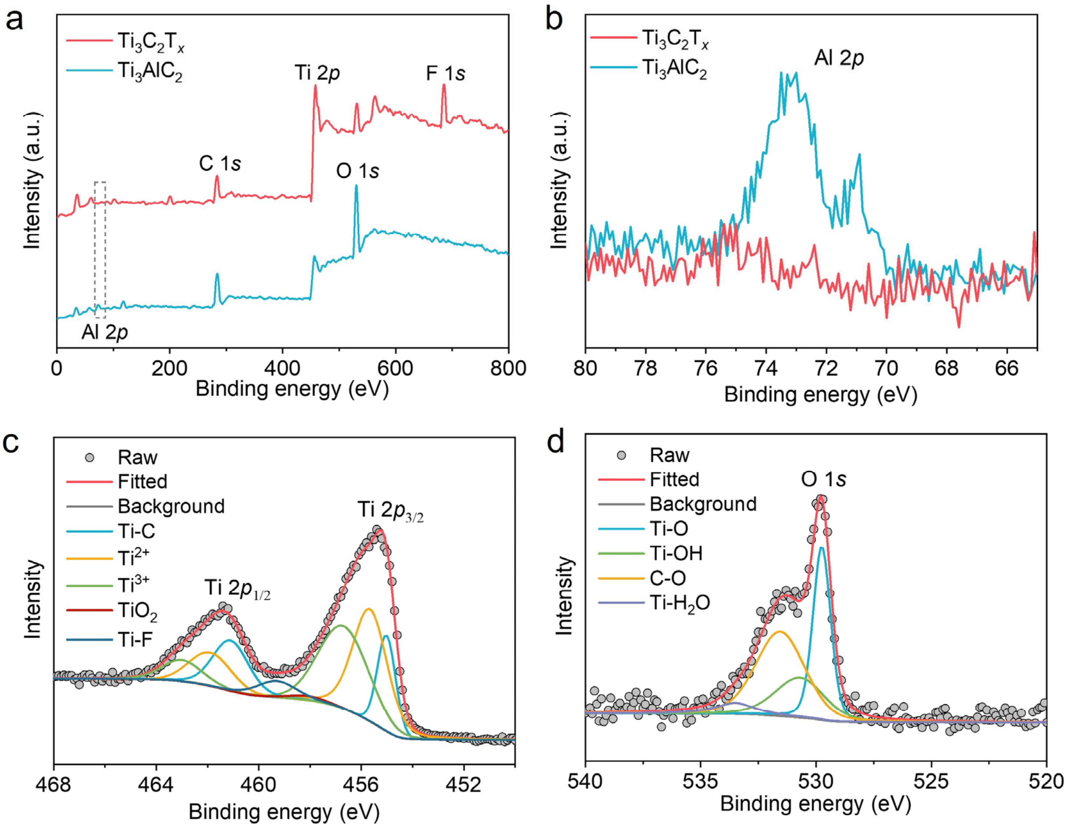

Supplementary Fig. 3 Elemental composition and surface groups of $Ti_3C_2T_x$ nanosheets. (a) XPS survey spectra and (b) Al 2*p* spectra of Ti_3AlC_2 and $Ti_3C_2T_x$ nanosheets. (c) Ti 2*p* and (d) O 1*s* XPS spectra for as-synthesized $Ti_3C_2T_x$ nanosheets.

Four surface terminations of $Ti_3C_2T_x$ nanosheets were obtained using LiF and HCl etching methods: Ti–O (type I), Ti–OH (type II), Ti–F (type III), and Ti–(H_2O)ads (type IV) [1]. In the Ti 2*p* spectra, the main peak of Ti $2p_{3/2}$ can be fitted with five peaks: 455.0 eV (Ti–C), 455.7 eV (Ti^{2+} for types I, II, and IV), 456.8 eV (Ti^{3+} for types I, II, and IV), 458.0 eV (TiO_2), and 459.3 eV (Ti–F for type III)[2]. The peak intensity of TiO_2 is almost negligible, indicating that the nanosheets were not oxidized during synthesis. In the O 1*s* spectra, the main peak can be fitted with four peaks at 529.8 eV (Ti–O), 530.7 eV (Ti–OH), 531.6 eV (C–O), and 533.5 eV (Ti–H_2O). Lewis acid Ti sites, which have empty orbitals, are common on the surface of $Ti_3C_2T_x$ MXene and are temporarily occupied by water molecules to form Ti–H_2O in aqueous solutions[3, 4].

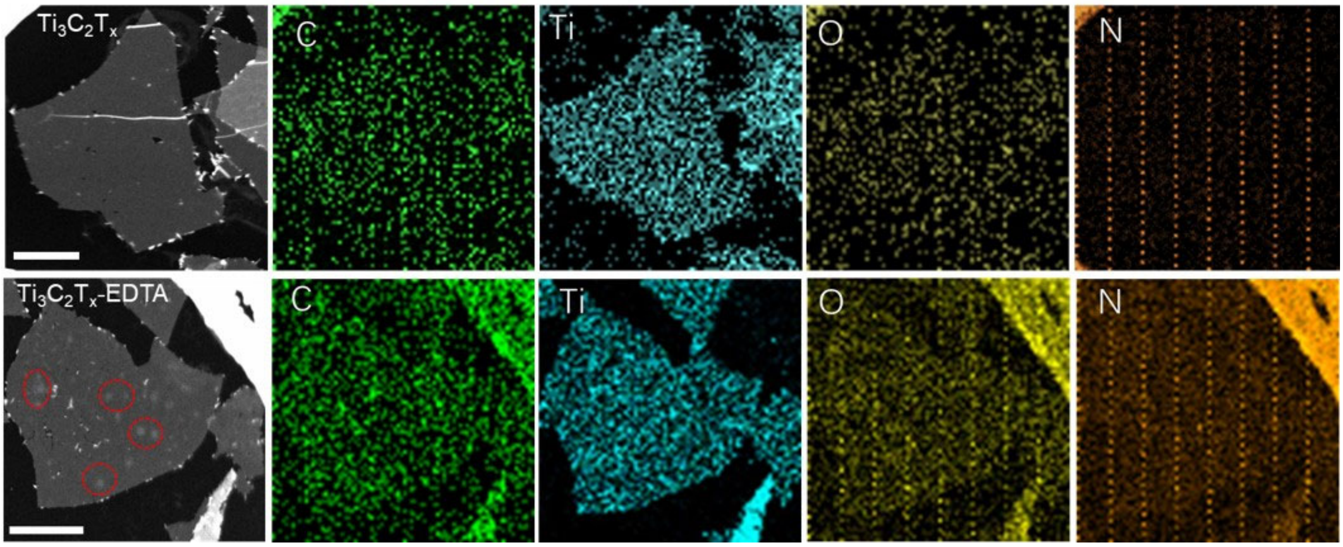

Supplementary Fig. 4 HAADF images and corresponding element mappings of $Ti_3C_2T_x$ and $Ti_3C_2T_x$-EDTA nanosheet. Scale bar, 500 nm.

As shown in HAADF image, obvious white patches (the circled position) appeared on the surface of $Ti_3C_2T_x$ nanosheets after mixing with EDTA solution. Together with the clear nitrogen signal in the element mapping image, it can be concluded that EDTA molecules were successfully grafted onto the $Ti_3C_2T_x$ nanosheets surface after the mixing process.

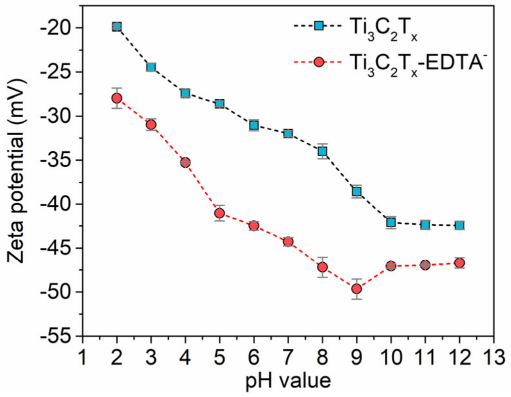

Supplementary Fig. 5 Zeta potential of $Ti_3C_2T_x$ and $Ti_3C_2T_x$-EDTA dispersions. Error bars represent the standard deviation of three measurements of a sample.

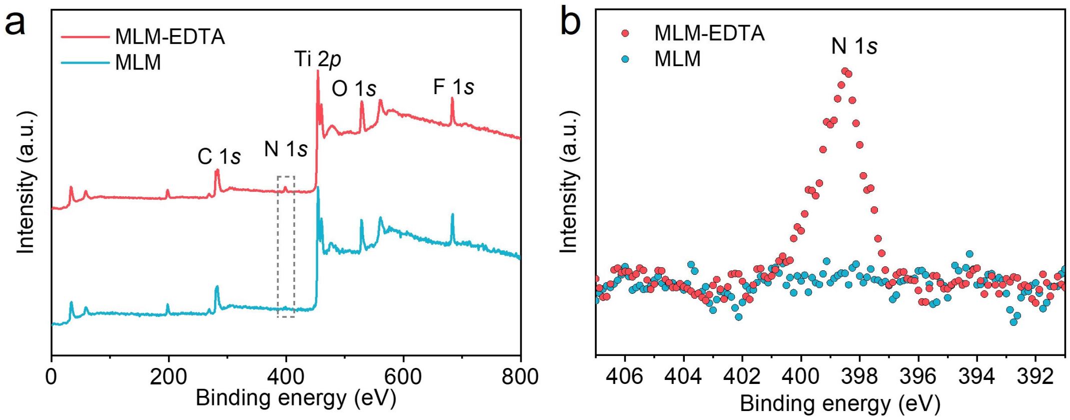

Supplementary Fig. 6 Successful intercalation of EDTA molecules. (a) XPS survey spectra and (b) N 1*s* spectra of MLM and MLM-EDTA membrane.

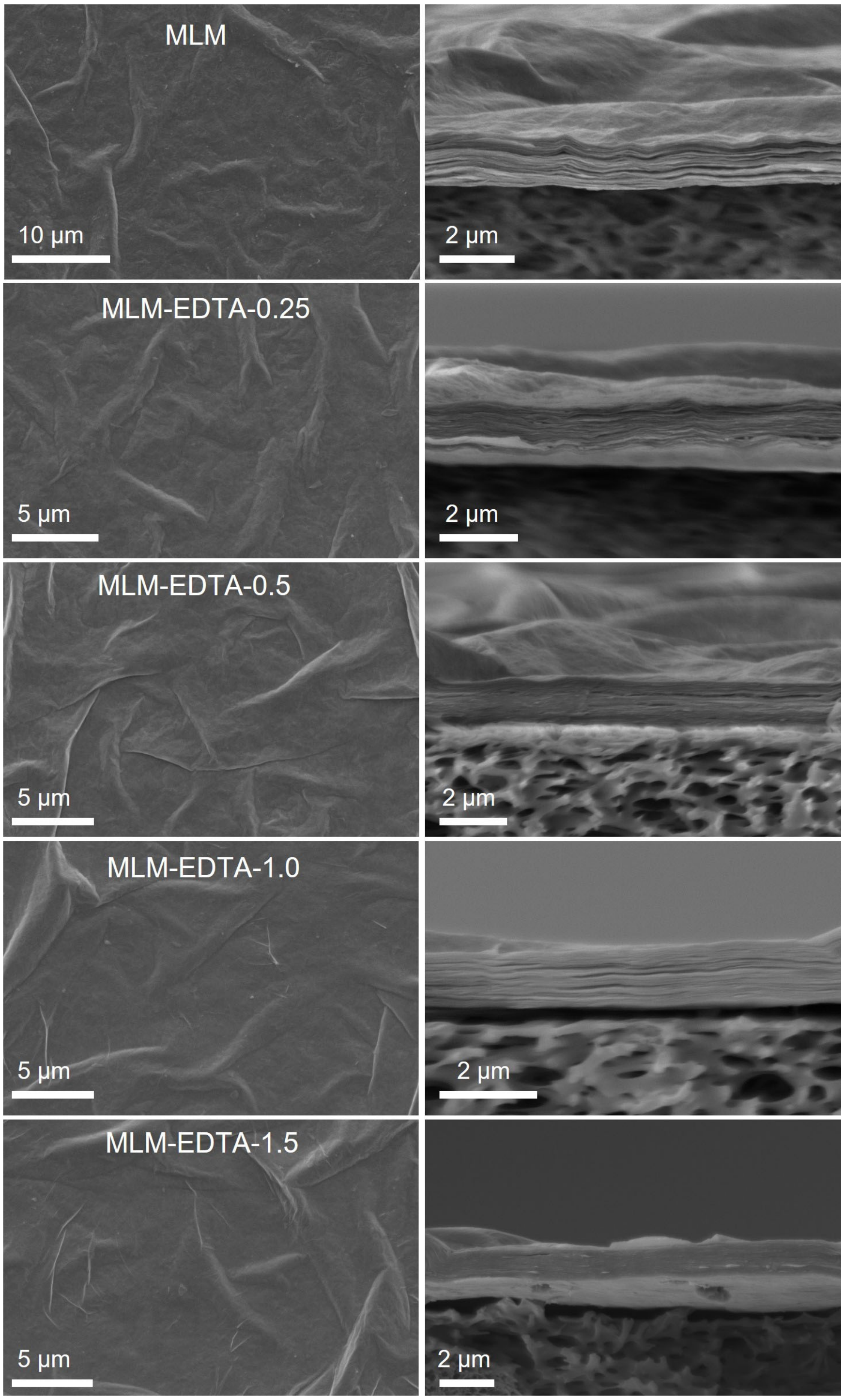

Supplementary Fig. 7 Surface SEM images (left) of MLM-EDTA-X (X represents the concentration of EDTA solution, X = 0, 0.25, 0.5, 1.0, 1.5 mg/mL) and corresponding cross-section images (right).

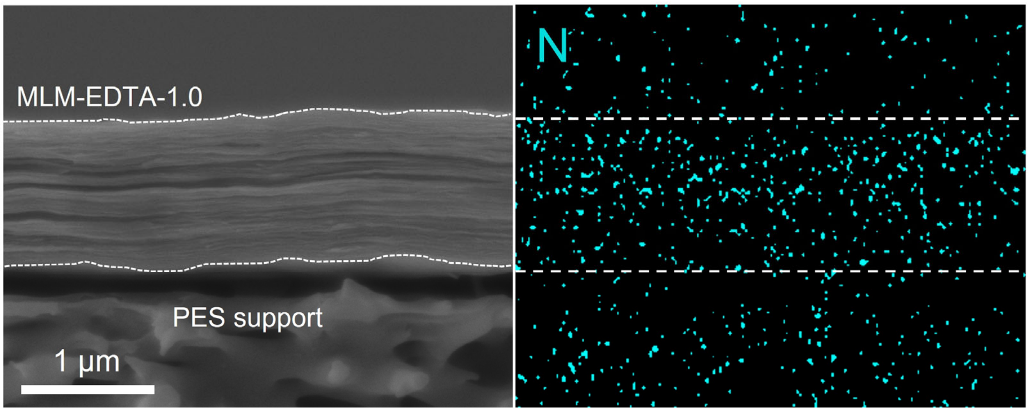

Supplementary Fig. 8 SEM image and corresponding element (N) mappings of the cross-section of an MLM-EDTA-1.0 membrane.

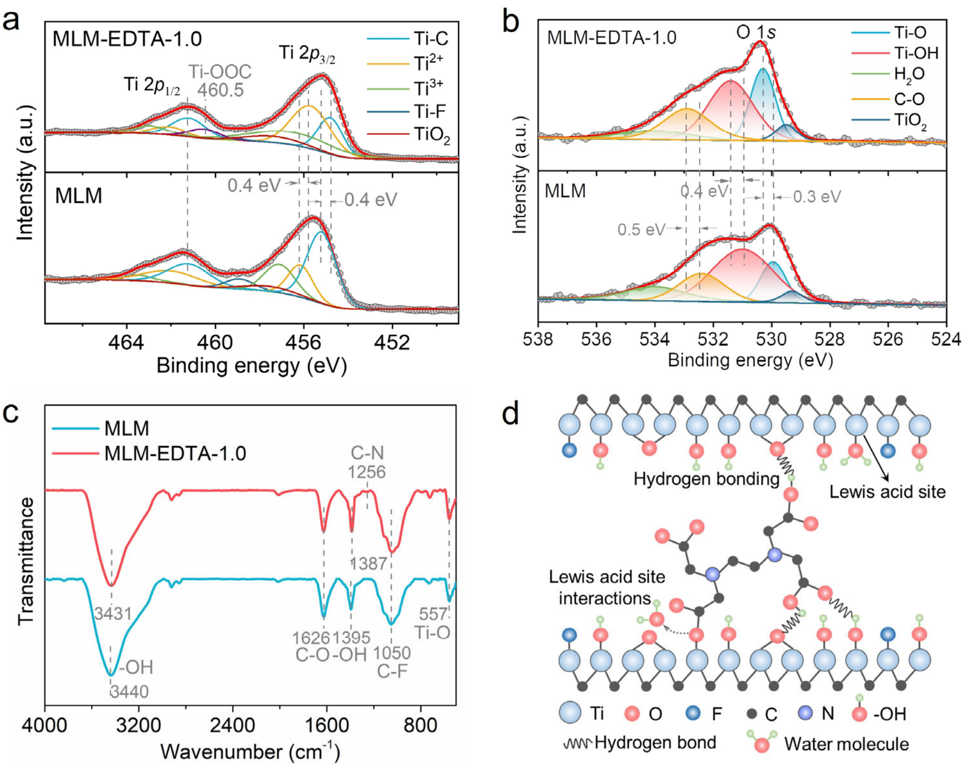

Supplementary Fig. 9 Interaction mechanism of EDTA molecules with $Ti_3C_2T_x$ nanosheets. (a) Ti 2*p*, (b) O 1*s* XPS spectra and (c) FT-IR spectra for MLM and MLM-EDTA-1.0. (d) Schematic diagram of the cross-linking mechanism between $Ti_3C_2T_x$ nanosheets and EDTA molecule.

The surface terminations of $Ti_3C_2T_x$ nanosheets obtained by LiF and HCl etching method comprise four possible types, including Ti–O (type I), Ti–OH (type II), Ti–F (type III) and Ti–$(H_2O)_{ads}$ (type IV). In the Ti 2*p* spectra of MLM, the main peak of Ti 2$p_{3/2}$ can be fitted by five peaks at 455.2 (Ti–C), 456.15 (Ti^{2+} (type I, II, IV)), 457.1 (Ti^{3+} (type I, II, IV)), 457.6 (TiO_2), and 458.9 (Ti–F, type III) eV[1, 2]. After thermal cross-linking with EDTA molecules, the Ti–C 2$p_{3/2}$ and Ti^{2+} (type I, II, IV) 2$p_{3/2}$ peaks are downshifted from 455.2 and 456.15 eV for MLM to 454.8 and 455.7 eV for MLM-EDTA-1.0 membrane, respectively. Because that oxygen atoms covalently bonded to Ti atoms normally have higher electronegativity, the the outer electron cloud density of Ti atoms will be decreased to lead to weakened shielding effect[5]. Moreover, a new peak appears at 460.5 eV due to the coordination between Ti atoms and carboxyl from EDTA molecules. In the O 1*s* spectra of MLM, the main peak of O 1*s* can be fitted by five peaks at 529.0 (TiO_2), 529.8 (Ti–O), 530.9 (Ti–OH), 532.4 (C–O) eV, 533.5 (Ti–H_2O)[2]. After cross-linking with EDTA molecules, the Ti–O, Ti–OH and C–O peaks are upshifted from 529.8, 530.9 and 532.4 eV for MLM to 530.3, 531.4 and 532.9 eV for MLM-EDTA-1.0, respectively. These changed indicate that Ti–O and Ti–OH are the active sites of EDTA molecules. FT-IR spectra show that compared with MLM, MLM-EDTA-1.0 has a new peak at 1256 cm^{-1} for the antisymmetric stretching vibration mode of C–N[6, 7], indicating the presence of EDTA molecules. Furthermore, the peak for stretching vibration of –OH is redshifted from 3,440 cm^{-1} for the MLM to 3,431 cm^{-1} for the MLM-EDTA, proving the hydrogen bonding between EDTA molecules and MXene nanosheets[5].

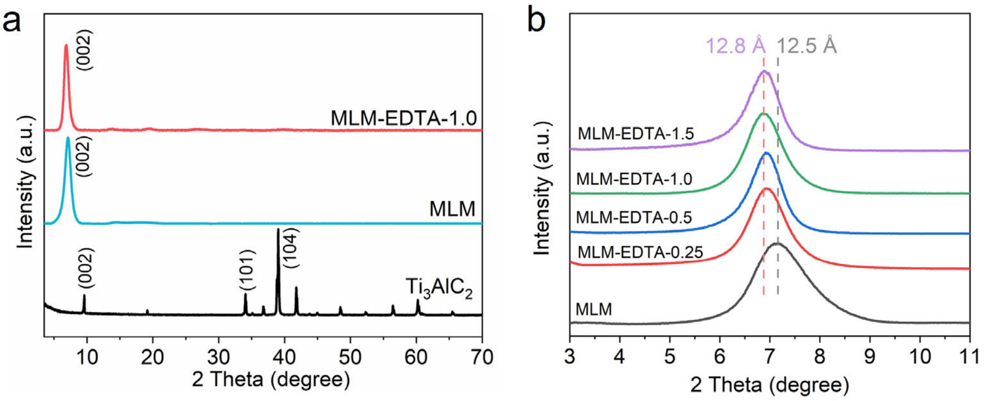

Supplementary Fig. 10 Crystal structure characterization of MXene membranes. (a) XRD patterns of the Ti_3AlC_2 powder, MLM and MLM-EDTA-1.0. (b) XRD patterns of MLM-EDTA-X in dry state (X represents the concentration of EDTA solution, X = 0, 0.25, 0.5, 1.0, 1.5 mg/mL).

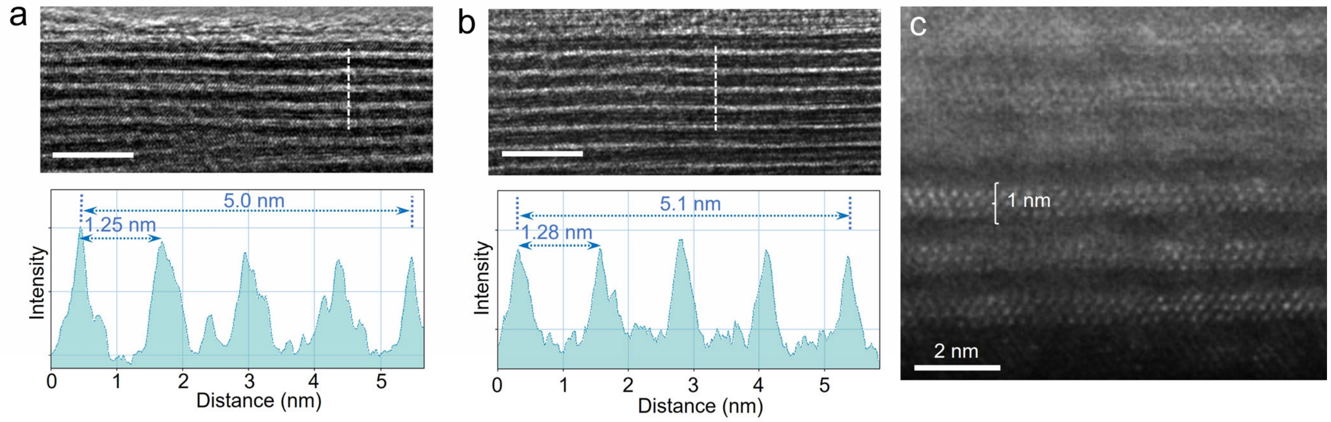

Supplementary Fig. 11 Interlayer structure of MXene membranes. High-resolution transmission electron microscopy (HRTEM) image of the cross-section of (a) MLM, (b) MLM-EDTA-1.0 and the corresponding interlayer spacing between neighboring nanosheets taken along the line. Scale bar, 5 nm. (c) STEM image of the cross-section of the MLM-EDTA-1.0.

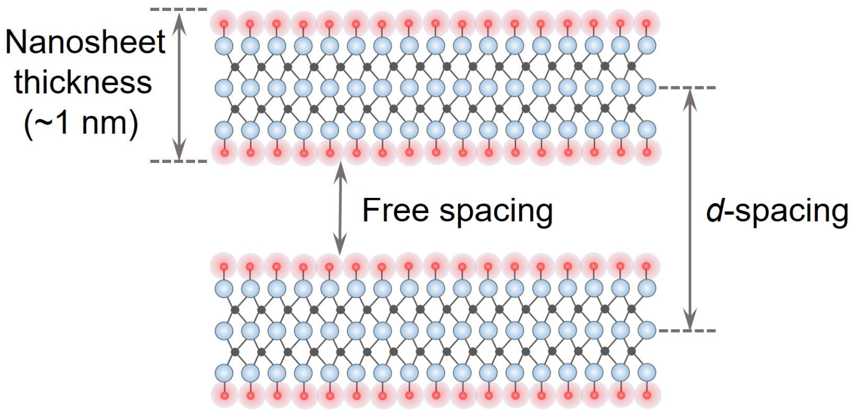

Supplementary Fig. 12 Schematic diagram of MLM laminar membrane free spacing calculation.

The d-spacing of the MLM laminar membrane was calculated based on the [002] peak position in the XRD pattern according to Bragg's law[8]. The d-spacing calculated via this method includes one $Ti_3C_2T_x$ layer plus one free spacing, where the thickness of the monolayered $Ti_3C_2T_x$ nanosheet has a thickness of 10.0 Å[9].

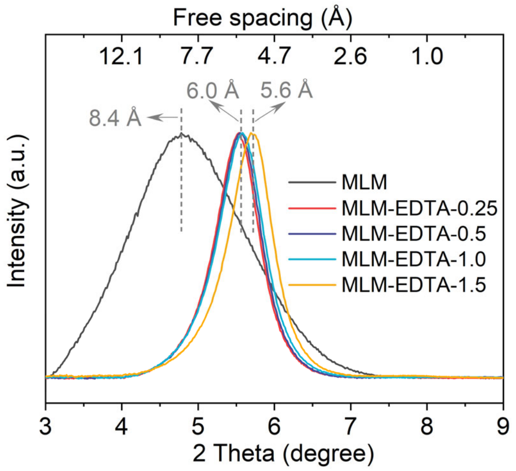

Supplementary Fig. 13 XRD patterns of MLM-EDTA-X (X represents the concentration of EDTA solution, X = 0, 0.25, 0.5, 1.0, 1.5 mg/mL) in mixed salts solution (KCl, NaCl, LiCl, $CaCl_2$ and $MgCl_2$).

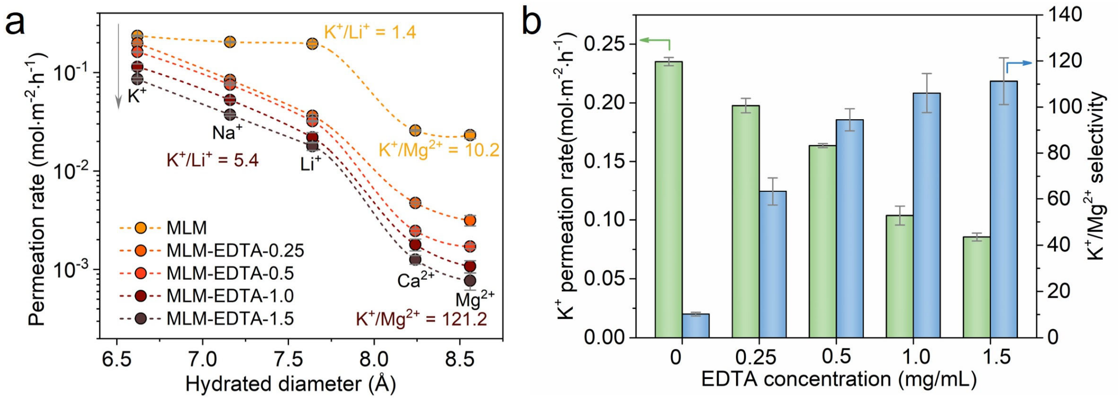

Supplementary Fig. 14 Ions separation capacity of MLM-EDTA-X membranes (X represents the concentration of EDTA solution, X = 0, 0.25, 0.5, 1.0, 1.5 mg/mL). (a) Ion permeation rates through MLM-EDTA-X membranes with different EDTA modification concentrations. (b) EDTA concentration-dependent K^+ permeation rates and K^+/Mg^{2+} selectivity of MLM-EDTA-X membranes. Error bars represent the standard deviation of three measurements of a sample.

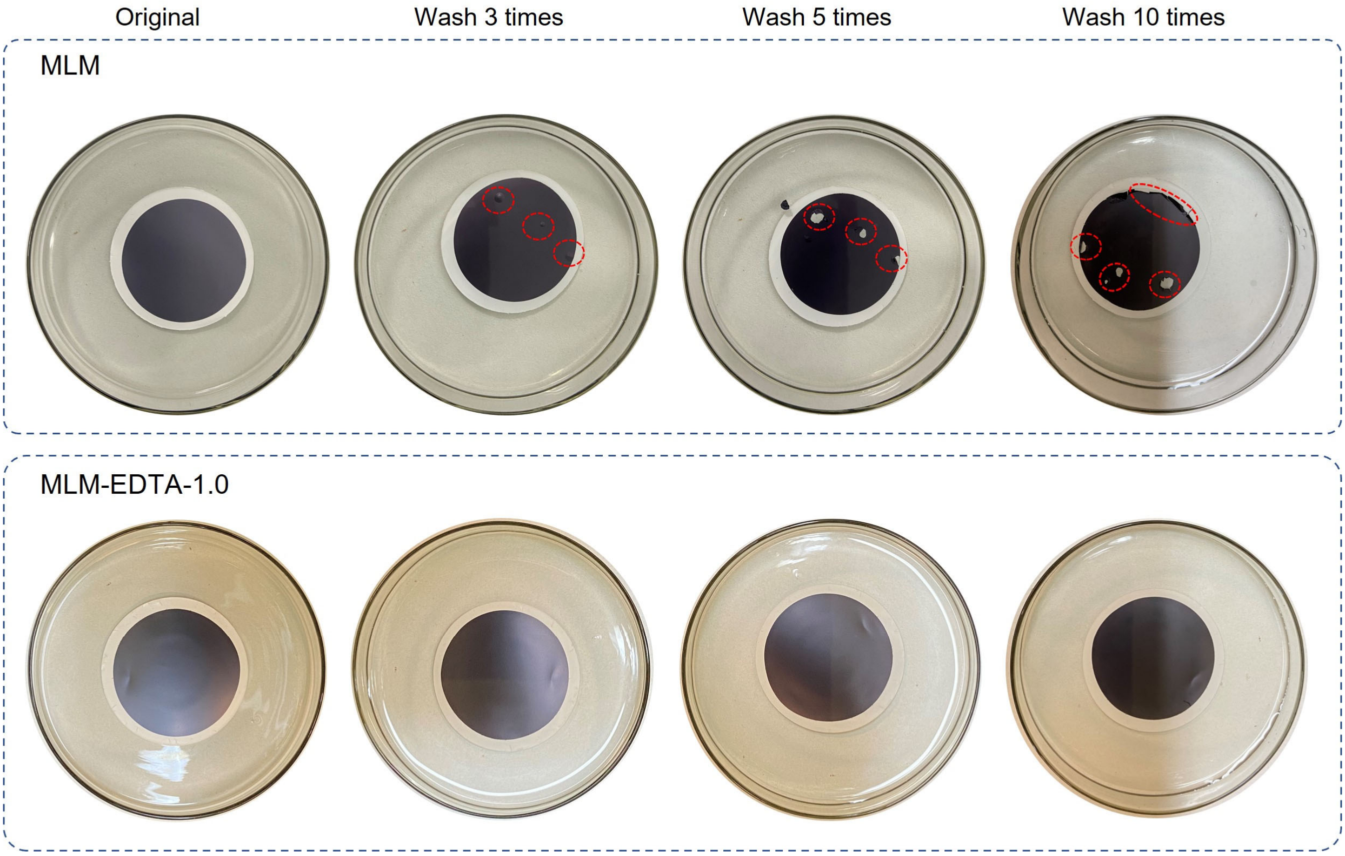

Supplementary Fig. 15 Optical images of MLM and MLM-EDTA-1.0 after several washes with mixed salt (KCl, NaCl, LiCl, $CaCl_2$ and $MgCl_2$) solution. Photo credit: Rongming Xu, Nanjing University.

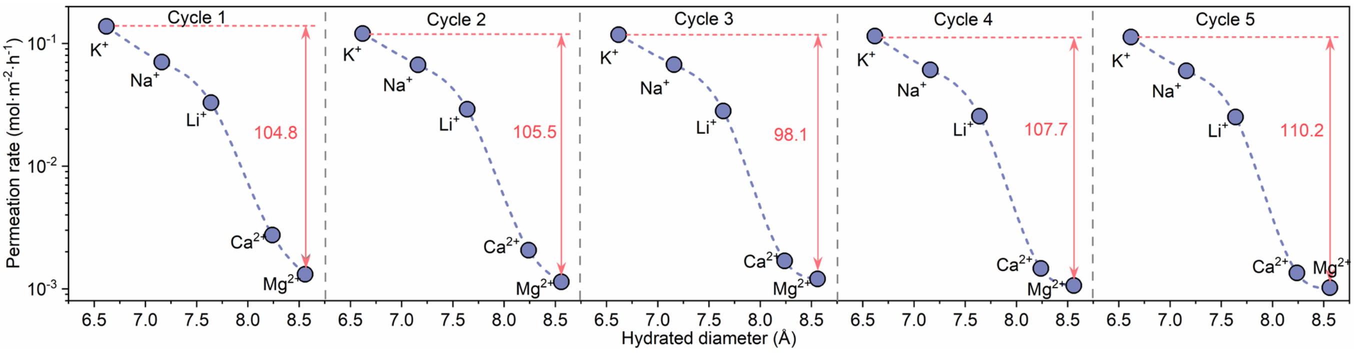

Supplementary Fig. 16 Cycling performance of MLM-EDTA-1.0.

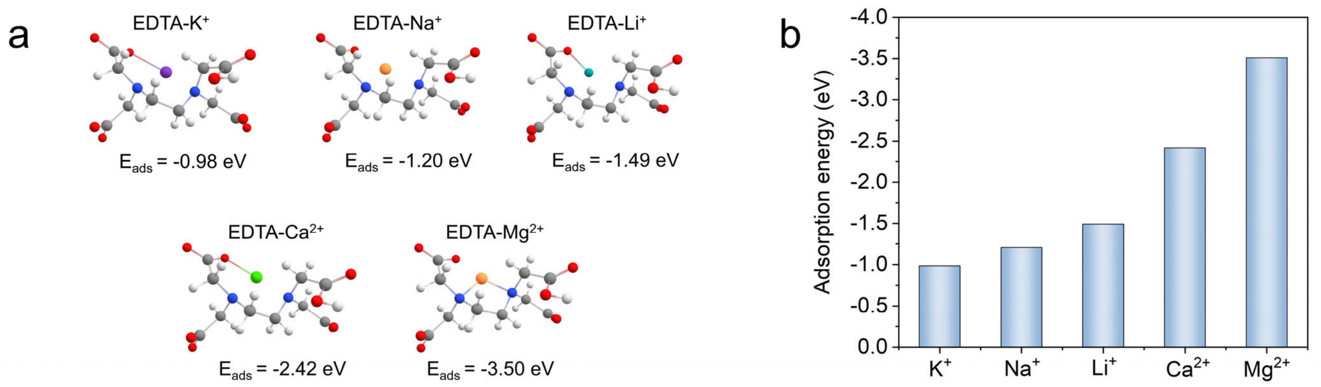

Supplementary Fig. 17 The adsorption energy between an EDTA molecule and various cations, calculated by Gaussian 09 software package. (a) Configuration diagrams of cationic adsorption with EDTA molecule after structural optimization. (b) The adsorption energy between the EDTA molecule and the cations.

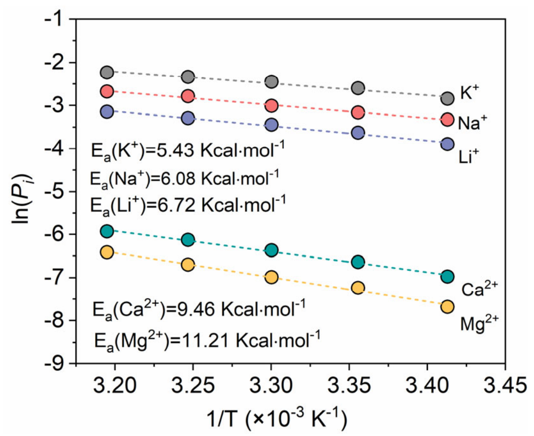

Supplementary Fig. 18 Arrhenius plots for cations diffusion through MLM-EDTA-1.5 in a 0.2 M mixed salts solution (KCl, NaCl, LiCl, $CaCl_2$ and $MgCl_2$) at pH 8.0.

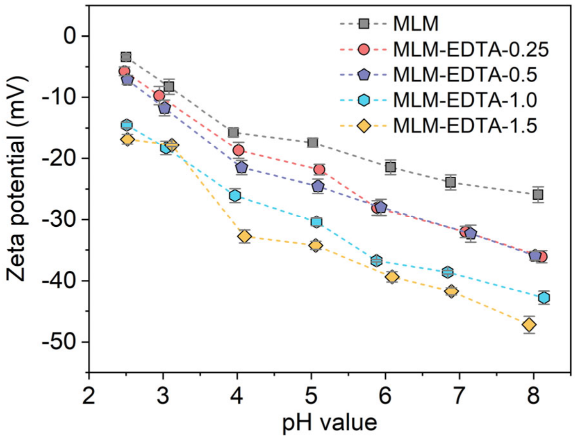

Supplementary Fig. 19 Zeta potential of MLM-EDTA-X. Error bars represent the standard deviation of three measurements of a sample.

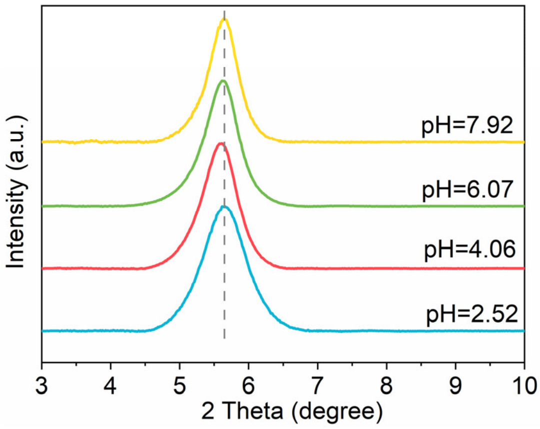

Supplementary Fig. 20 XRD patterns of MLM-EDTA-1.5 in different pH values mixed salts (KCl, NaCl, LiCl, $CaCl_2$ and $MgCl_2$) solutions.

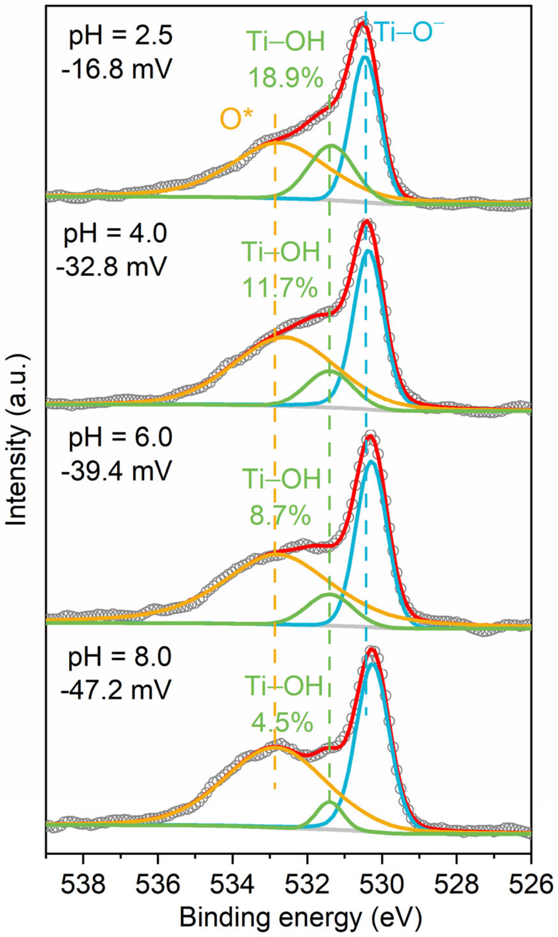

Supplementary Fig. 21 O 1*s* XPS spectra for MLM-EDTA-1.5 in mixed salts solution at different pH.

The main peak of O 1*s* can be fitted by four peaks at 528.8 (TiO_2), 530.3 (Ti–O), 531.5 (Ti–OH), 532.8 (O*, include C–O and Ti–H_2O) eV[1, 2]. The percentage of Ti–OH decreases continuously from 18.9% to 4.5 %with the pH value from 2.5 to 8.0, which indicates that the source of negative charge inside of the channel is mainly the deprotonation of Ti–OH.

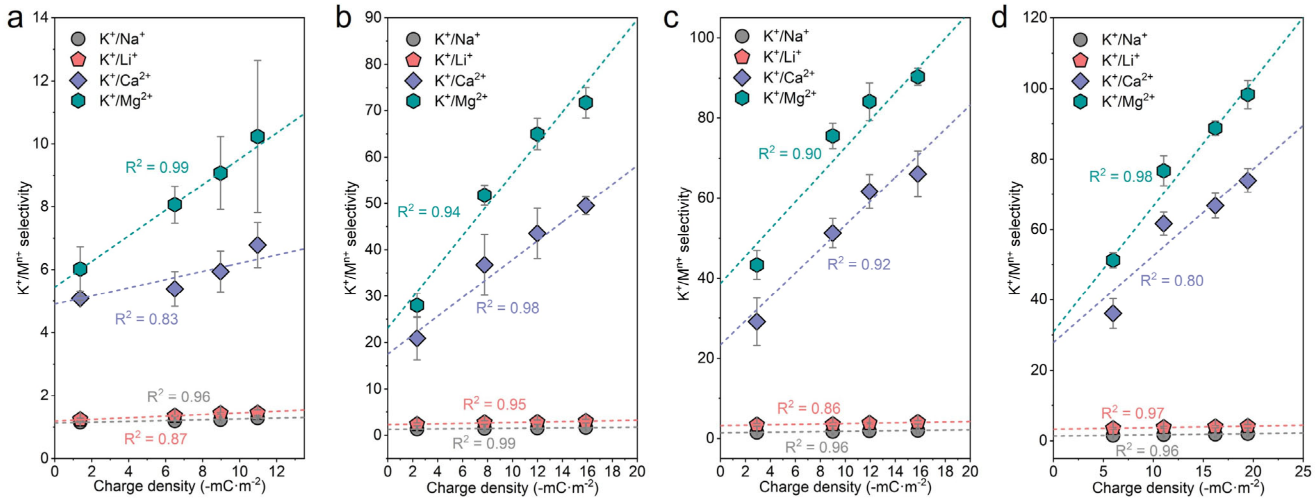

Supplementary Fig. 22 Correlation between charge density and ions selectivity. Relationship between charge density and K^+/M^{n+} (M^{n+} is Na^+, Li^+, Ca^{2+}, Mg^{2+}, respectively) selectivity for (a) MLM, (b) MLM-EDTA-0.25, (c) MLM-EDTA-0.5, (d) MLM-EDTA-1.0. Error bars represent the standard deviation of three measurements of a sample.

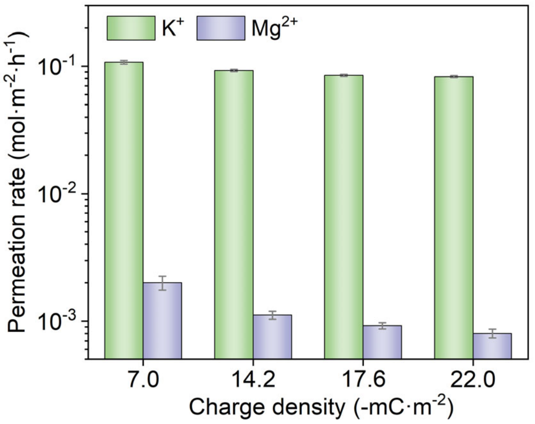

Supplementary Fig. 23 K^+ and Mg^{2+} permeation rates through MLM-EDTA-1.5 under different charge densities. Error bars represent the standard deviation of three measurements of a sample.

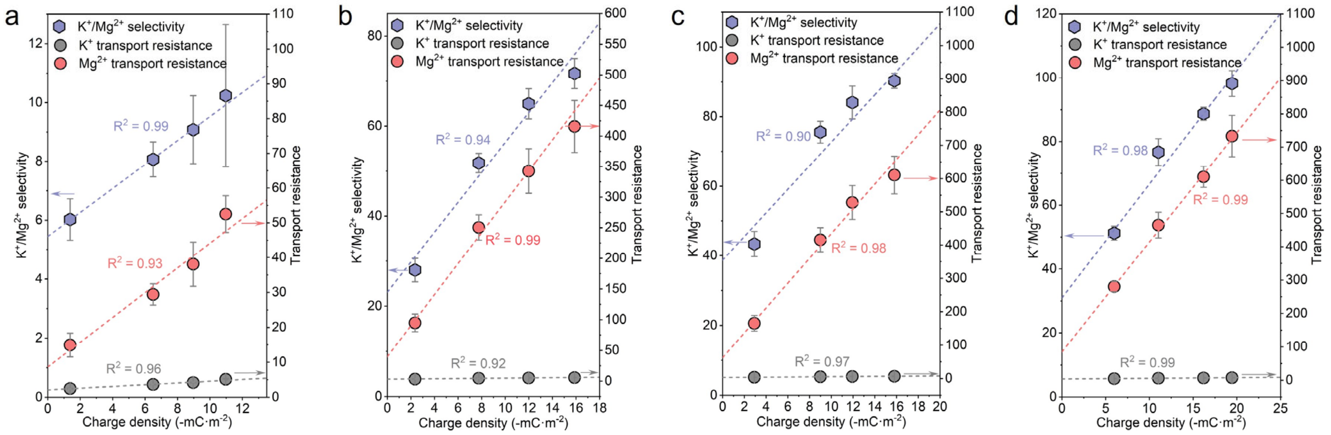

Supplementary Fig. 24 Correlation between charge density and ions transport resistance. Relationship between K^+, Mg^{2+} transport resistance (Reciprocal of ion permeation rates, $1/P_i$) and K^+/Mg^{2+} selectivity with charge density as independent variable for (a) MLM, (b) MLM-EDTA-0.25, (c) MLM-EDTA-0.5, (d) MLM-EDTA-1.0. For the transport resistance data, the intersection of linear fits with y-axis represents the transport resistance caused by other effects except the charge effect. Error bars represent the standard deviation of three measurements of a sample.

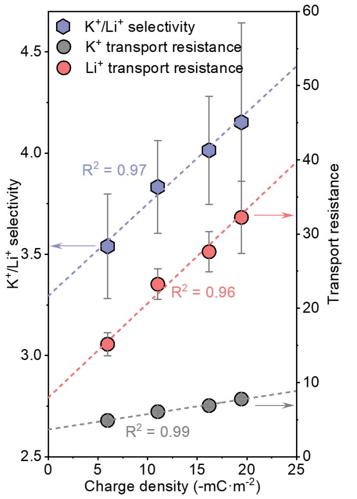

Supplementary Fig. 25 Relationship between K^+, Li^+ transport resistance (Reciprocal of ion permeation rates, $1/P_i$) and K^+/Li^+ selectivity with charge density as independent variable for MLM-EDTA-1.0. Error bars represent the standard deviation of three measurements of a sample.

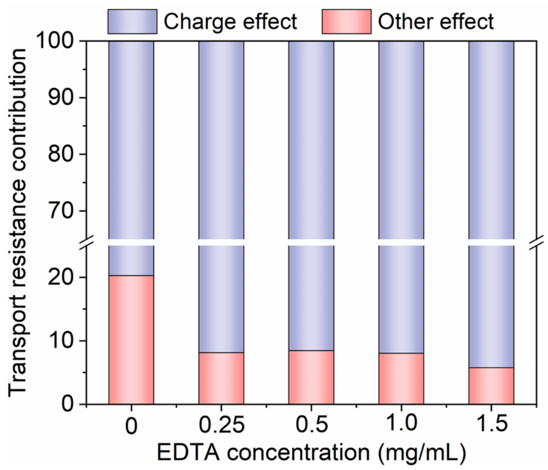

Supplementary Fig. 26 The transport resistance contribution (obtained by Supplementary Fig. 24) of charge effect or other effect for Mg^{2+} passing through MLM-EDTA-X membranes at pH of 8.0.

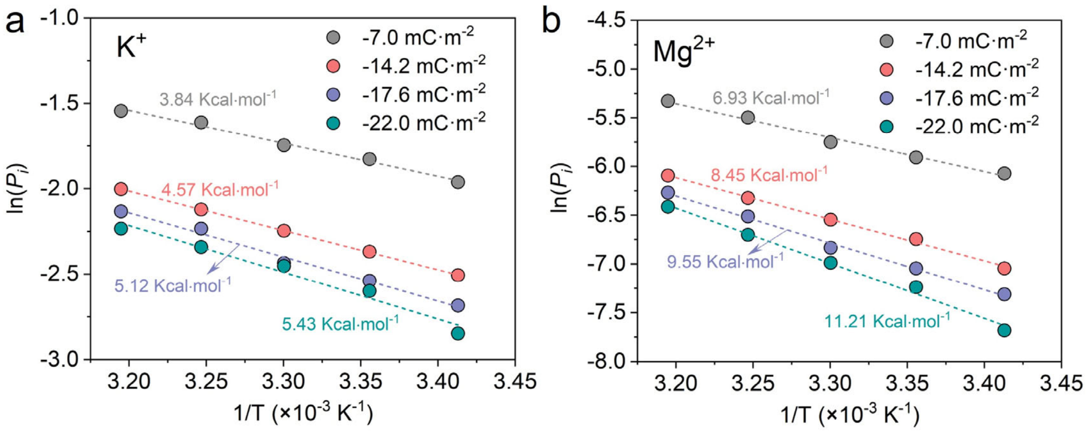

Supplementary Fig. 27 Energy barriers for ions diffusion through MLM-EDTA-1.5 under different charge densities. Arrhenius plots for (a) K^+ and (b) Mg^{2+} diffusion through MLM-EDTA-1.5 in a 0.2 M mixed salt solution at different charge densities.

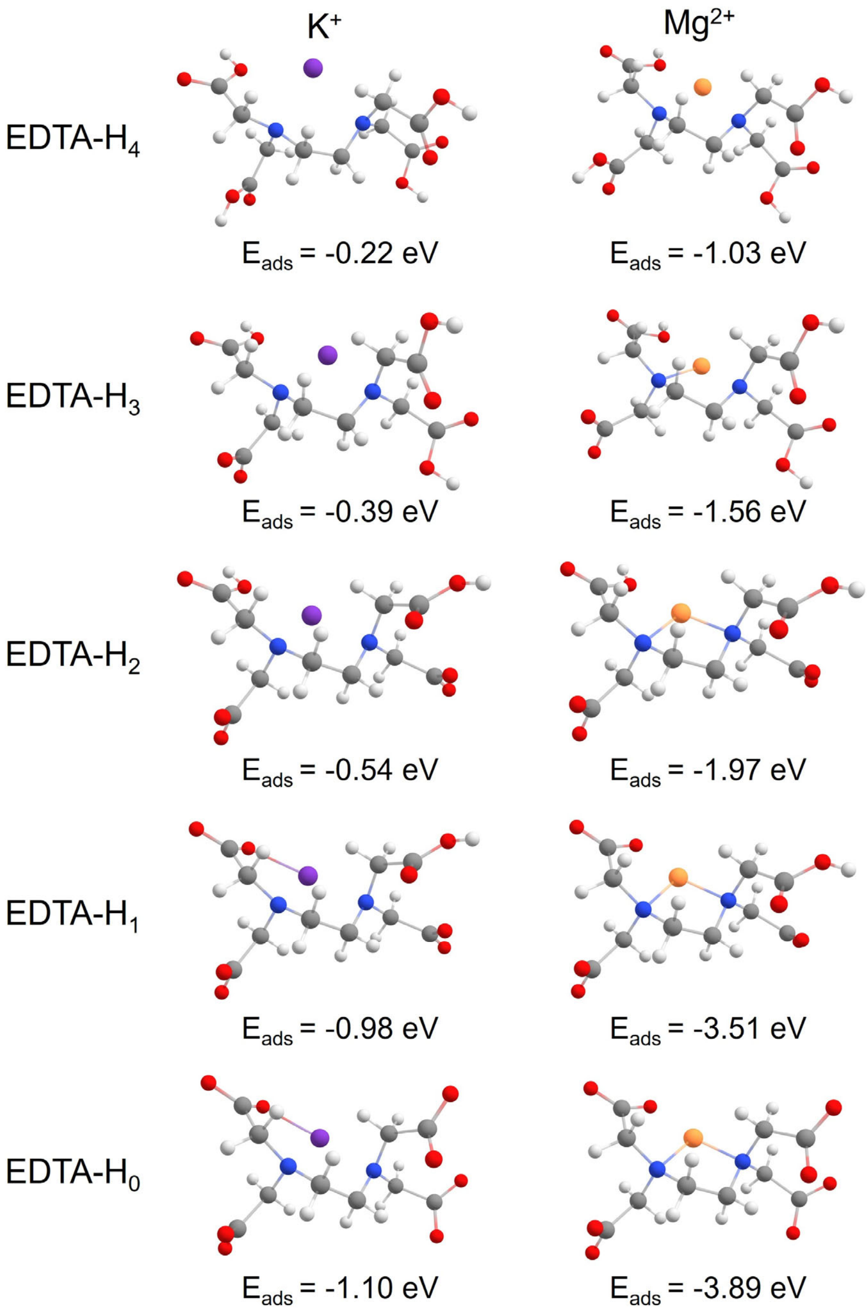

Supplementary Fig. 28 DFT calculated adsorption energy for K^+ and Mg^{2+} on EDTA molecules with different negatively charged oxygen atoms, calculated by Gaussian 09 software package.

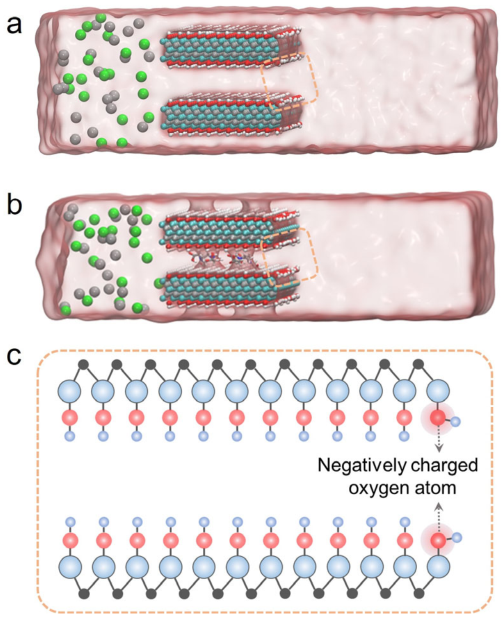

Supplementary Fig. 29 Schematic diagram of MD simulation model. Snapshots of MD simulation (obtained by VMD software package, version 1.9.3[10]) for K^+ permeation through the (a) MLM and (b) MLM-EDTA at 0 ns. (c) Schematic diagram of exposed negatively charged oxygen atoms. K^+ and Cl^- ions are in green and silver, respectively. A row of oxygen atoms at the exit of the channel were exposed, otherwise the ion permeation rate would be below the detect limit[11, 12].

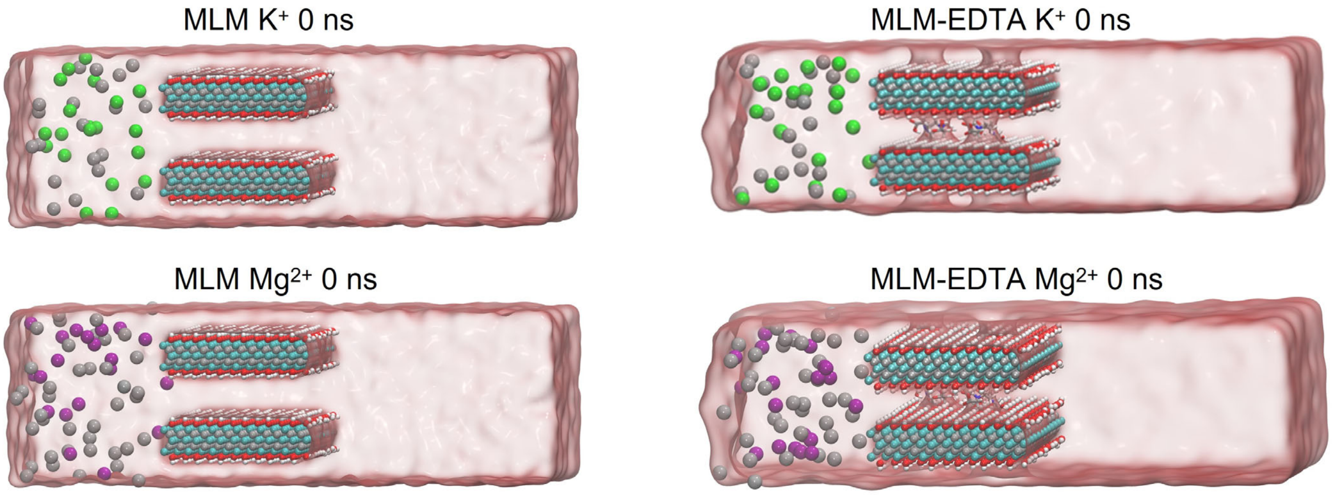

Supplementary Fig. 30 Snapshots of MD simulation for K^+ and Mg^{2+} passing the MLM (interlayer spacing = 18.4 Å) and MLM-EDTA (interlayer spacing = 16.0 Å) systems at 0 ns. K^+, Mg^{2+} and Cl^- ions are in green, purple and silver, respectively.

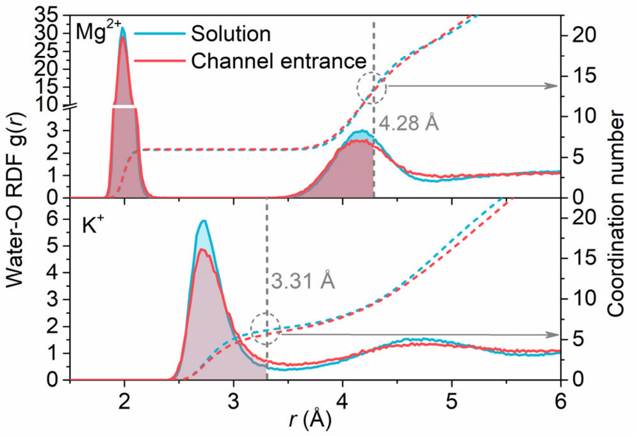

Supplementary Fig. 31 Radial distribution function (RDF) of oxygen in water molecules around K^+ and Mg^{2+} locating at the solution and MLM channel entrance. Dashed lines represent the coordination number of water molecules. The valid range of the data was taken from 0 to the hydration radius of the ions (3.31 Å for K^+ and 4.28 Å for Mg^{2+}). The hydration numbers of K^+ and Mg^{2+} were hardly changed when they entry the MLM channel.

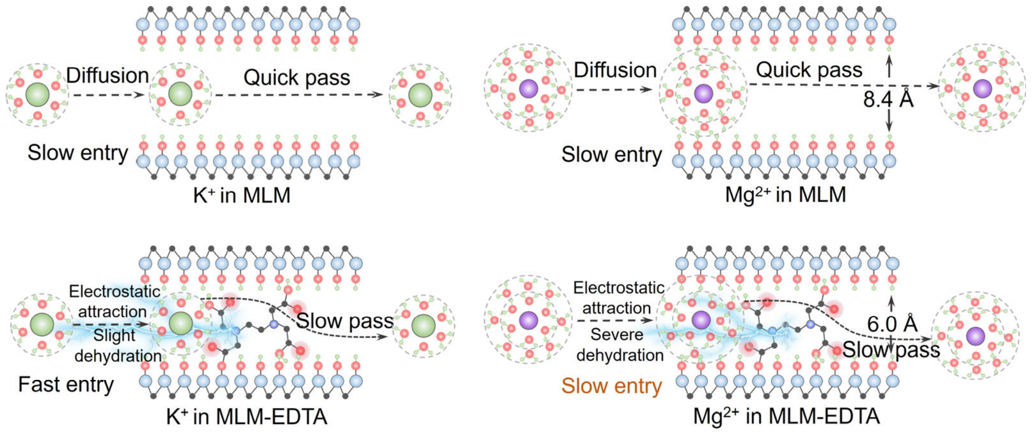

Supplementary Fig. 32 Schematic diagram of the mechanism for EDTA molecules enhances ion selectivity.

The decoration of EDTA molecules amplifies the time gap between K^+ and Mg^{2+} entering the channel, resulting in significantly higher K^+/Mg^{2+} selectivity of the MLM-EDTA membrane than that of MLM.

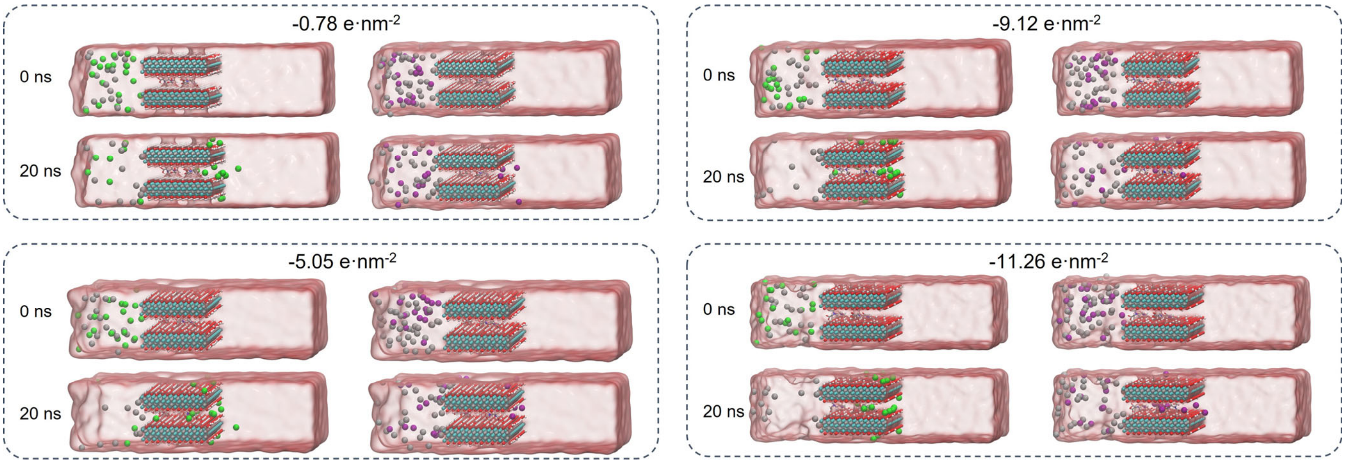

Supplementary Fig. 33 Snapshots of MD simulation (obtained by VMD software package, version 1.9.3) for K^+ and Mg^{2+} passing the MLM-EDTA channel (interlayer spacing = 16.0 Å) with different charge densities at 0 and 20 ns. K^+, Mg^{2+} and Cl^- ions are in green, purple and silver, respectively.

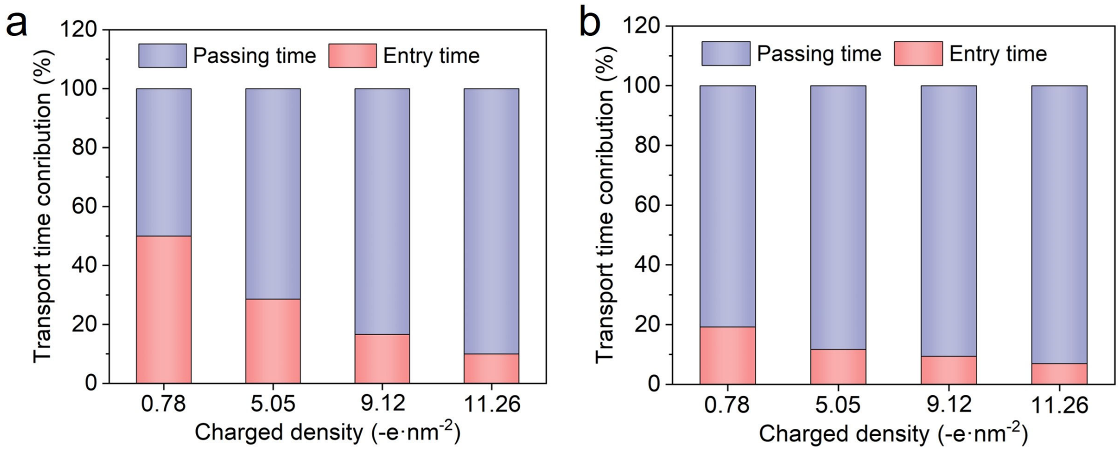

Supplementary Fig. 34 Comparison of the elapsed time for ions to enter and pass through the MLM-EDTA channel at different charge densities. The contribution by percentage (obtained from Figure. 4e) of entry time and passing time to the overall transport time for (a) Mg^{2+} and (b) K^{+} passing through MLM-EDTA with different charge densities.

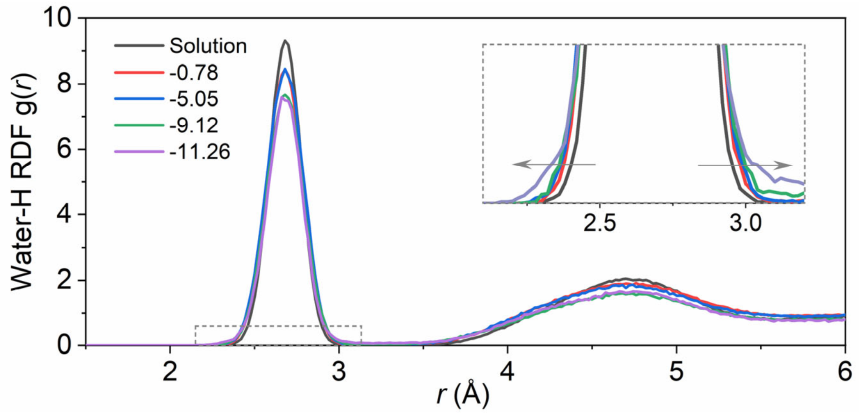

Supplementary Fig. 35 RDF of hydrogen in water molecules around Mg^{2+} locating at the solution and inside of MLM-EDTA channel, the channel with different charge densities.

The width of the first shell increases with an increasing negative charge density, which corresponds to an increased number of negatively charged oxygen atoms. When the channel is narrow enough to be comparable to the diameter of hydrated ions, hydrogen atoms readily form hydrogen bonds with oxygen atoms on the inner wall of the channel. As a result, water molecules around Mg^{2+} are rotated and moved outward due to the increased hydrogen bonding.

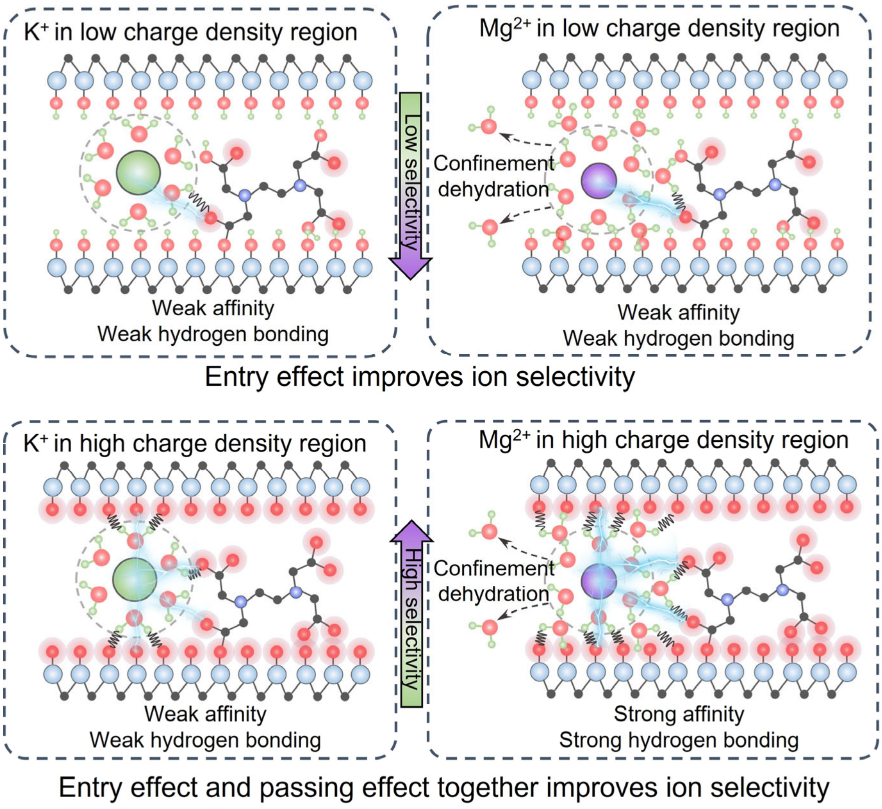

Supplementary Fig. 36 Schematic illustration of the mechanism by which charge density enhances ion selectivity.

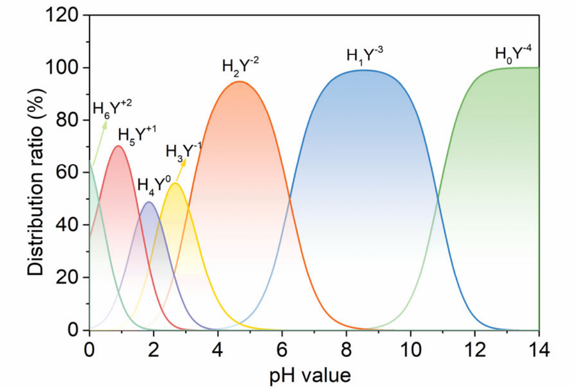

Supplementary Fig. 37 The distribution of EDTA molecule in different pH solution.

The distribution curve of EDTA was calculated by Visual MINTEQ 3.1 software package, T=25 °C. Depending on the number of charges, EDTA ($C_{10}H_{16}N_2O_8$) can be written as H_tY^n. Due to the easy protonation of the 2 nitrogen atoms on EDT under strong acidic conditions, and the active nature of the 4 carboxyl groups, the value of *t* is 0, 1, 2, 3, 4, 5, 6. Corresponding value of *n* is -4, -3, -2, -1, 0, +1, +2, respectively.

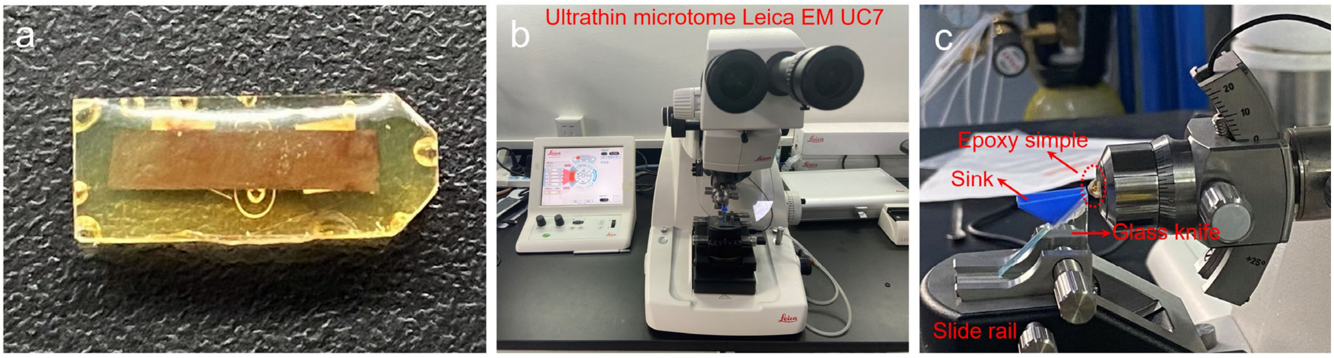

Supplementary Fig. 38 Sample preparation for TEM characterization for membrane cross-sections. Optical image of (a) resin-embedded membrane sample, (b) ultrathin microtome, (c) slicing process details. Photo credit: Rongming Xu, Nanjing University.

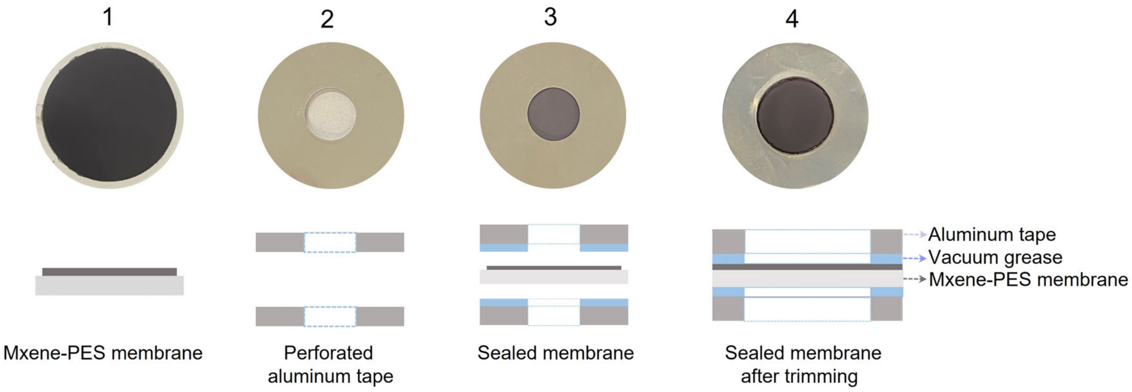

Supplementary Fig. 39 Schematic diagram of membrane encapsulating.

First, fold the aluminum tape in half, ensuring that the holes are perfectly aligned, and then punch a round hole with a diameter of 16 mm. Second, apply vacuum grease evenly to the inside of the aluminum tape, and then carefully sandwich the membrane between the two aluminum tapes. Finally, flatten and trim the sealed membrane to a suitable size. Photo credit: Rongming Xu, Nanjing University.

Supplementary Table 1 Properties of cations.

Ions	Bare diameter (Å)[13]	Hydrated diameter (Å)[13]	−ΔHyd$G$* (kJ·mol^{-1})[14]
K^+	2.66	6.62	295
Na^+	1.90	7.16	365
Li^+	1.20	7.64	475
Ca^{2+}	1.98	8.24	1505
Mg^{2+}	1.30	8.56	1830

Supplementary Table 2 Performance comparison of K^+/Mg^{2+} selectivity vs K^+ permeation rates for various

membranes from concentration difference-driven diffusion measurements.

Membrane	Ion channel dimension[a] (Å)	Measurement system	Ion concentration	Selectivity (K^+/Mg^{2+})	K^+ permeation rate ($mol \cdot m^{-2} \cdot h^{-1}$)	Standardized K^+ permeation rate[b]	Ref.
GO membrane							
GO	10	Single salt solution	1 M	~1.1	2	2	15
i(0.5)-GO	8.4	Single salt solution	0.5 M	7.96	1.15	2.3	16
i(1.0)-GO	8.52	Single salt solution	0.5 M	9.11	1.36	2.72	16
i(1.5)-GO	8.57	Single salt solution	0.5 M	8.63	1.36	2.72	16
i(2.0)-GO	8.61	Single salt solution	0.5 M	8.7	1.61	3.22	16
GO/LDH-NS(Co-Al)	7.7	Single salt solution	0.1 M	~4.5	~0.18	1.8	17
GO/LDH-NS(Mg-Al)	-	Single salt solution	0.1 M	~11	~0.21	2.1	17
Pristine-GO	8.18	Mixed salts solution	0.1 M	3.37	0.064	0.64	18
EDA-GO	7.0	Mixed salts solution	0.1 M	3.5	0.068	0.68	18
PDA-GO	6.85	Mixed salts solution	0.1 M	4.2	0.092	0.92	18
BDA-GO	6.93	Mixed salts solution	0.1 M	5.0	0.107	1.07	18
HMDA-GO	6.29	Mixed salts solution	0.1 M	4.4	0.118	1.18	18
PPD-GO	6.2	Mixed salts solution	0.1 M	7.15	0.088	0.88	18
OPD-GO	5.64	Mixed salts solution	0.1 M	6.7	0.066	0.66	18
TA-GO	6.7	Mixed salts solution	0.05 M	18.89	0.017	0.34	19
TA-GO	6.7	Mixed salts solution	0.1 M	14.87	0.056	0.56	19
TA-GO	6.7	Mixed salts solution	0.2 M	13.90	0.08	0.4	19
PA GO	~7.55	Mixed salts solution	0.1 M	4.85	0.034	0.34	20
PA/EDA GO	~7.85	Mixed salts solution	0.1 M	14.4	0.072	0.72	20
PA/PDA GO	~7.85	Mixed salts solution	0.1 M	5.28	0.037	0.37	20
PA/BDA GO	~7.6	Mixed salts solution	0.1 M	3.75	0.03	0.3	20
PA/HMDA GO	~7.8	Mixed salts solution	0.1 M	5.6	0.028	0.28	20
PA/PPD GO	~5.96	Mixed salts solution	0.1 M	10	0.01	0.1	20
PA/OPD GO	~7.24	Mixed salts solution	0.1 M	5.3	0.016	0.16	20
$Ti_3C_2T_x$ membrane							
$Ti_3C_2T_x$	6.4	Single salt solution	0.2 M	~6	0.9	4.5	8
Al_{13}–$Ti_3C_2T_x$ (11.5)	1.5	Single salt solution	0.5 M	~3	0.012	0.024	21
$Ti_3C_2T_x$/PSS	5.9	Single salt solution	0.2 M	~2.3	0.006	0.03	22
EPD-$Ti_3C_2T_x$	4.8	Single salt solution	0.2 M	~1.8	0.008	0.04	23
Ca-SAT-$Ti_3C_2T_x$	6.5	Single salt solution	0.5 M	~4	0.2	0.4	24
Al^{3+}-$Ti_3C_2T_x$	5.2	Single salt solution	0.2 M	~2.5	0.0015	0.0075	12
Polymer membrane							

CNT-CMP polymer	8.4	Single salt solution	0.01 M	~2	0.1	1	[25]
Prussian blue polymer	-	Single salt solution	0.1 M	10	0.042	0.42	[26]
Prussian white polymer	-	Single salt solution	0.1 M	28.57	0.12	1.2	[26]
PIM-EA-TB	4-10	Single salt solution	1 M	~10	10	10	[27]
PIM-BzMA-TB	4-9	Single salt solution	1 M	~26.6	1.2	1.2	[27]
DMBP-TB	4.5-9	Single salt solution	1 M	~50	0.2	0.2	[27]
PVDF-PIM	-	Single salt solution	0.1 M	1.84	0.036	0.36	[28]
GZ-PIM4	-	Single salt solution	0.1 M	7.27	0.024	0.24	[28]
GZ-PIM3	-	Single salt solution	0.1 M	9.68	0.032	0.32	[28]
GZ-PIM2	-	Single salt solution	0.1 M	9.38	0.032	0.32	[28]
GZ-PIM1	-	Single salt solution	0.1 M	9.33	0.03	0.3	[28]
This work							
MLM-EDTA-1.5	5.6	Mixed salts solution	0.2 M	121.2	0.085	0.43	This work

Since the ion separation performance obtained by different driving methods is not comparable[29], only the reported values under concentration difference-driven diffusion are listed.

[a] The membrane ion channel dimension is the free spacing distance of the membrane in the wet state.

[b] For fairness, K^+ permeation rates are standardized by KCl initial concentration.

Supplementary Reference

1. Halim, J. et al. X-ray photoelectron spectroscopy of select multi-layered transition metal carbides (MXenes). *Appl. Surf. Sci.* **362**, 406-417 (2016).
2. Ding, L. et al. MXene molecular sieving membranes for highly efficient gas separation. *Nat. Commun.* **9**, 155 (2018).
3. Ding, L. et al. Bioinspired $Ti_3C_2T_x$ MXene-Based Ionic Diode Membrane for High-Efficient Osmotic Energy Conversion. *Angew. Chem. Int. Ed.* **61**, e202206152 (2022).
4. Zhang, Z. et al. Mechanically strong MXene/Kevlar nanofiber composite membranes as high-performance nanofluidic osmotic power generators. *Nat. Commun.* **10**, 2920 (2019).
5. Wan, S. et al. High-strength scalable MXene films through bridging-induced densification. *Science* **374**, 96-99 (2021).
6. Peng, Y. et al. A versatile MOF-based trap for heavy metal ion capture and dispersion. *Nat. Commun.* **9**, 187 (2018).
7. Boota, M. et al. Pseudocapacitive Electrodes Produced by Oxidant-Free Polymerization of Pyrrole between the Layers of 2D Titanium Carbide (MXene). *Adv. Mater.* **28**, 1517-1522 (2016).
8. Ren, C.E. et al. Charge- and Size-Selective Ion Sieving Through $Ti_3C_2T_x$ MXene Membranes. *J. Phys. Chem. Lett.* **6**, 4026-4031 (2015).
9. Wang, J. et al. Ion sieving by a two-dimensional $Ti_3C_2T_x$ alginate lamellar membrane with stable interlayer spacing. *Nat. Commun.* **11**, 3540 (2020).
10. Humphrey, W. et al. VMD: Visual Molecular Dynamics. *J. Mol. Graph.* **14**, 33-38 (1996).
11. Abraham, J. et al. Tunable sieving of ions using graphene oxide membranes. *Nat. Nanotechnol.* **12**, 546-550 (2017).
12. Ding, L. et al. Effective ion sieving with $Ti_3C_2T_x$ MXene membranes for production of drinking water from seawater. *Nat. Sustain.* **3**, 296-302 (2020).
13. Nightingale, E.R. Phenomenological theory of ion solvation. Effective radii of hydrated ions. *J. Phys. Chem.* **63**, 1381-1387 (1959).
14. Marcus, Y. A simple empirical model describing the thermodynamics of hydration of ions of widely varying charges, sizes, and shapes. *Biophys. Chem.* **51**, 111-127 (1994).
15. Joshi, R. K. et al. Precise and Ultrafast Molecular Sieving Through Graphene Oxide Membranes. *Science* **343**, 752-754 (2014).
16. Zhang, M. et al. Designing Biomimic Two-Dimensional Ionic Transport Channels for Efficient Ion Sieving. *ACS nano* **15**, 5209-5220 (2021).
17. Sun, P. et al. Highly selective charge-guided ion transport through a hybrid membrane consisting of anionic graphene oxide and cationic hydroxide nanosheet superlattice units. *NPG Asia Materials* **8**, e259-e259 (2016).
18. Jia, Z. et al. Diamines cross-linked graphene oxide free-standing membranes for ion dialysis separation. *J. Membr. Sci.* **520**, 139-144 (2016).
19. Lim, M.-Y. et al. Cross-linked graphene oxide membrane having high ion selectivity and antibacterial activity prepared using tannic acid-functionalized graphene oxide and polyethyleneimine. *J. Membr. Sci.* **521**, 1-9 (2017).
20. Jia, Z. & Shi, W. Tailoring permeation channels of graphene oxide membranes for precise ion separation. *Carbon* **101**, 290-295 (2016).
21. Zhu, J. et al. Precisely Tunable Ion Sieving with an Al_{13}-$Ti_3C_2T_x$ Lamellar Membrane by Controlling Interlayer Spacing. *ACS nano* **14**, 15306-15316 (2020).
22. Lu, Z. et al. A Lamellar MXene ($Ti_3C_2T_x$)/PSS Composite Membrane for Fast and Selective Lithium-Ion Separation. *Angew. Chem. Int. Ed.* **60**, 22265-22269 (2021).
23. Deng, J. et al. Fast electrophoretic preparation of large-area two-dimensional titanium carbide membranes for ion sieving. *Chem. Eng. J.* **408**, 127806 (2021).
24. Wang, P. et al. Ultrafast ion sieving using nanoporous polymeric membranes. *Nat. Commun.* **9**, 569 (2018).
25. Zhou, Z. et al. Precise Sub-Angstrom Ion Separation Using Conjugated Microporous Polymer Membranes. *ACS nano* (2021).

26. Li, Z. et al. Reversible Electrochemical Tuning of Ion Sieving in Coordination Polymers. *Anal. Chem.* **92**, 9172-9178 (2020).
27. Tan, R. et al. Hydrophilic microporous membranes for selective ion separation and flow-battery energy storage. *Nat. Mater.* **19**, 195-202 (2020).
28. Kazemzadeh, H. et al. Evaluation of polymer inclusion membrane efficiency in selective separation of lithium ion from aqueous solution. *Sep. Purif. Technol.* **251**, 117298 (2020).
29. Wang, R. et al. Understanding Selectivity in Solute-Solute Separation: Definitions, Measurements, and Comparability. *Environ. Sci. Technol.* **56**, 2605-2616 (2022).
